# A deep Siamese network framework for precision phage selection in pulmonary infections

**DOI:** 10.3389/fmed.2026.1758028

**Published:** 2026-02-04

**Authors:** Xinghong Wang, Mingpeng Fu, Shigang Lin, Yanshuang Wang, Hua Pei

**Affiliations:** 1 Department of Clinical Laboratory, The Second Affiliated Hospital of Hainan Medical University, Haikou, China; 2 School of Computer Science and Technology, Hainan University, Haikou, China

**Keywords:** artificial intelligence, phage selection, phage therapy, pulmonary infection, Siamese network

## Abstract

Pulmonary infections pose a significant global health challenge to human life and health. In patients with chronic pulmonary diseases such as cystic fibrosis and bronchiectasis, structural abnormalities of the airways and impaired mucociliary clearance contribute to recurrent and challenging pulmonary infections. These infections are frequently complicated by antimicrobial resistance, making them difficult to treat with conventional antibiotics. As a result, phage therapy has emerged as a promising alternative for treating resistant pulmonary infections. Recently, the integration of artificial intelligence (AI) has improved the efficiency of phage selection. Nevertheless, the accuracy of predicting phage–bacterial host interactions remains limited, posing a significant obstacle to the clinical translation of phage-based therapies. To address this issue, we propose a deep Siamese network framework for precision phage selection in pulmonary infections. Specifically, we employ an identical model architecture to process both phage and host genomes. Initially, the genomic sequences of both phages and hosts are encoded into feature representations using k-mer segmentation followed by the skip-gram model. Subsequently, convolutional neural networks (CNNs) and Transformers are introduced to extract local and global features, respectively. Finally, the extracted features are fused to predict phage–host interactions. Experimental results on dataset created from the NCBI genome database demonstrate that our proposed method achieves superior performance in the precise identification of phages targeting specific bacterial hosts, thereby supporting its potential application in phage therapy for pulmonary infections.

## 1 Introduction

Pulmonary infections, particularly those involving the lower respiratory tract, represent a major global public health challenge and pose a substantial threat to human health. Lower respiratory tract infections are among the most lethal infectious diseases worldwide and rank as the fourth leading cause of death (1). Their incidence and severity are notably elevated in patients with chronic pulmonary diseases, such as cystic fibrosis (CF) and bronchiectasis, in whom abnormal airway architecture and impaired mucociliary clearance create a favorable niche for persistent bacterial colonization and proliferation. In individuals with CF, progressive pulmonary disease remains the primary cause of morbidity and mortality, with recurrent and chronic infections serving as a dominant driver of pathological deterioration (2, 3). Currently, antibiotics remain a relatively effective treatment for pulmonary infections, however, the emergence of antibiotic resistance has made the treatment of pulmonary infections more complex and difficult. The World Health Organization has listed antibiotic-resistant bacteria as one of the three major public health threats of the 21st century (1). In 2019, antibiotic-resistant diseases directly caused 1.27 million deaths and indirectly caused 4.95 million deaths, with pneumonia and other lower respiratory tract infections being the leading causes of death, accounting for 1.9 million deaths. More worryingly, if the current trend continues, by 2050, diseases caused by antibiotic-resistant bacteria could cause 10 million deaths annually (1, 4). Among the multitude of drug-resistant bacteria, the ESKAPE pathogens—Enterococcus faecium, Staphylococcus aureus, Klebsiella pneumoniae, Acinetobacter baumannii, Pseudomonas aeruginosa, and Enterobacter species—are particularly noteworthy for their ability to “escape” conventional antibiotic treatments and rapidly proliferate in hospital settings (5). Among them, Carbapenem-resistant Acinetobacter baumannii (CRAB) is an important pathogen of pneumonia. Meanwhile, Pseudomonas aeruginosa is one of the most frequently isolated pathogens from CF patients, exhibiting resistance to multiple antibiotics (6, 7).

Faced with the increasingly severe challenge of antibiotic resistance, clinicians have had to rely on a few still-active antibiotics, such as minocycline, to treat CRAB pneumonia (6, 7). However, these "last-line" antibiotics also face the risk of future ineffectiveness. To date, the main challenges in treating pulmonary infections include: (1) biofilm formation, which reduces antibiotic permeability and efficacy; (2) the rapid development of bacterial resistance; (3) chronic infections requiring long-term treatment, increasing the risk of side effects; and (4) the destructive effects of antibiotics on the host microbiome. Therefore, the development of novel anti-infection strategies is urgently needed. Phage therapy has regained attention in recent years as an alternative treatment option (4, 8). Phages are viruses that naturally reside in bacteria, capable of infecting and killing their bacterial hosts, including antibiotic-resistant strains. Phage therapy offers several advantages: high specificity (it does not harm the host microbiota), self-replication capability (it multiplies at the site of infection), and potent anti-biofilm activity (5). Significant progress has been made in the treatment of drug-resistant pulmonary infections using this approach (5). Multiple preclinical studies and compassionate use cases have demonstrated the significant efficacy of phage therapy for infections that cannot be cured by other methods. Furthermore, phage-antibiotic synergy (PAS) has emerged as a promising strategy to overcome the limitations of monotherapy (1, 5).

Despite the immense potential of phage therapy, its widespread application faces numerous challenges. Traditional phage screening relies on empirical trial-and-error methods, a cumbersome and time-consuming process (9). Furthermore, bacterial resistance to phages evolves rapidly, primarily through mechanisms such as receptor blocking and CRISPR-Cas system activation (9, 10). To overcome these limitations, AI technology (11, 12) has been introduced into phage research, significantly improving screening efficiency. For instance, machine learning algorithms facilitate the prediction of phage–host interactions, enabling the identification of highly efficient phage–bacteria pairs (13). AI-assisted genome editing further provides powerful tools for tailoring phage properties (14). AI-based approaches also allow prediction of phage lifestyle (lytic vs. lysogenic) (9, 10). Nevertheless, current AI methods remain substantially limited in accurately forecasting phage–bacteria interactions, such as the inability to extract deeper characterizations, thus limiting their translational potential in clinical practice.

To address the above issue, we present a deep Siamese network framework for precision phage selection in pulmonary infections. Specifically, this framework utilizes a unified model architecture to process both phage and host genomes. First, the genomic sequences of both are encoded using the k-mer method. Then, CNN (15) and Transformers (16, 17) are employed to extract local and global features, respectively (18). Finally, the extracted features are integrated to predict phage–host interactions. Experimental results on a dataset created from the NCBI genome database demonstrate that our proposed method achieves state-of-the-art performance. Our proposed framework enables the screening of matching phages for specific bacterial hosts, thereby supporting phage therapy in pulmonary infections.

In summary, our contributions are as follows:

- We propose a deep Siamese network framework that efficiently screens phages matching specific bacterial hosts, thereby supporting the application of phage therapy in pulmonary infections.
- We incorporate CNN and Transformer architectures into the deep Siamese network to extract complementary local and global representations of phages and their hosts, thereby enabling more accurate prediction of phage–host interactions.

## 2 Related work

### 2.1 Phage-host interactions

Predicting phage–host interactions is fundamental to microbial ecology and phage therapy, and is essential for elucidating phage–host networks and identifying optimal therapeutic phages. Traditional approaches primarily rely on sequence alignment tools such as NCBI BLAST (19) to infer host relationships through homology searches. However, the high diversity and rapid evolution of phage genomes often lead to weak homology signals, making these methods insufficiently sensitive for metagenomic data, especially when dealing with short fragments or distant evolutionary relationships. To address these limitations, a series of alignment-free methods has been proposed. For example, oligonucleotide frequency–based approaches predict hosts by comparing differences in k-mer distributions (20), achieving high prediction accuracy for metagenomic viral sequences. Similarly, WIsH (21) applies a Markov model to predict hosts for phage contigs, while PHP (22) employs a Gaussian model to analyze prokaryotic virus sequences. By eliminating dependency on sequence alignment (23, 24), these methods enhance both robustness and computational efficiency. With the advancement of machine learning, protein feature–based methods have further improved prediction performance. For instance, RaFAH (25) accurately identifies bacterial and archaeal hosts by analyzing viral protein composition combined with a classifier model. HoPhage (26), a *de novo* prediction tool designed for metavirome fragments, integrates both sequence and protein features to effectively handle short viral contigs. In addition, network-based methods such as (27) integrate multiple data sources—including sequence similarity and co-occurrence networks—into a unified framework for systematically predicting virus–prokaryote interactions.

### 2.2 Application of AI in phage therapy

In addition to phage–host interaction prediction, AI is increasingly being applied to other key aspects of phage therapy, accelerating its transition from laboratory research to clinical practice. In the prediction of phage protein function, the tool PhageDPO employs a support vector machine model to identify depolymerases—encoded within phage genomes—that are capable of degrading bacterial polysaccharide structures, thereby offering novel targets for disrupting biofilm barriers (28). In the realm of phage genome design, the Prophage Activation platform utilizes a random forest model to predict regulatory elements in prophages and enables the activation of dormant prophages by simulating edits to transcription factor binding sites (13). This approach provides a new strategy for precise antimicrobial intervention from within drug-resistant bacteria. At the clinical level, AI is further harnessed to optimize phage cocktail formulations and monitor the emergence of bacterial resistance (29). Together, these applications underscore the considerable potential of AI technologies in advancing phage therapy from empirical screening toward a rational, design-based framework.

## 3 Method

### 3.1 Data pre-processing

This study adopted the dataset construction scheme consistent with VirHostMatcher-Net (27) to ensure comparability. We collected 2,288 phage genomes with known host information from the NCBI RefSeq database (accessed November 11, 2019). From these, 826 phages with specifically identified hosts at the strain level were selected to construct the positive training set, including 817 bacteria-infecting and 9 archaea-infecting phages. The remaining 1,462 phage-host pairs were held out as the final independent test set for all performance evaluations. Host information was extracted from the “isolate_host” or “host” fields in the GenBank records (27). For negative sample construction, we implemented a taxonomic distance-based sampling strategy by randomly selecting 826 phage-host pairs with the requirement that each selected host belongs to a different phylum from the phage's true host. While we acknowledge potential false negatives in these non-interacting pairs, the impact is minimized by the specificity of phage-host interactions and the phylum-level exclusion criterion (27).

### 3.2 Deep Siamese network framework

We propose a deep Siamese network framework to accurately predict phage-host interactions, thereby selecting a precise phage for the treatment of pulmonary infections. The model formulates the task as a binary classification problem (match/non-match), with the core idea being to learn high-level features from the complete genome sequences of both phages and hosts, and then evaluate the likelihood of their interaction.

**Figure 1:**
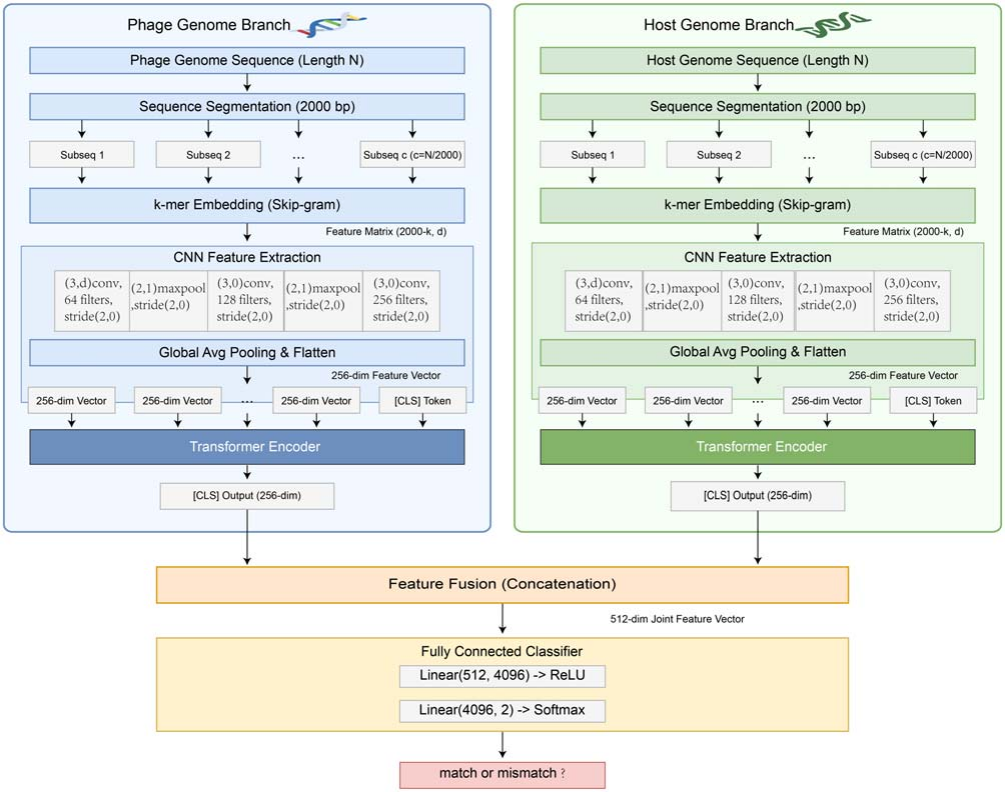
Overall architecture of the proposed Deep Siamese Network Framework. The framework consists of four core modules: (1) Sequence Preprocessing and Feature Representation module responsible for segmenting long genome sequences and converting them into numerical feature matrices; (2) Local Feature Learning module that uses CNN to extract local patterns from sequence segments; (3) Global Dependency Learning module that employs Transformer encoders to integrate all local features, forming a whole-genome representation; and (4) Fully Connected Classifier module for match prediction.

The overall architecture, as shown in Figure 1, consists of four core modules: a Sequence Preprocessing and Feature Representation module responsible for segmenting long genome sequences and converting them into numerical feature matrices; a Local Feature Learning module that uses CNN to extract local patterns from sequence segments; a Global Dependency Learning module that employs Transformer encoders to integrate all local features, forming a whole-genome representation; and a Fully Connected Classifier module for match prediction. Model training aims to minimize the cross-entropy loss function, optimizing all parameters via the backpropagation algorithm.

#### 3.2.1 Sequence preprocessing and feature representation

First, standardized preprocessing is applied to the input phage and host genome sequences.For a genome sequence of length *N* (where *N* is the genome sequence length in base pairs), due to the CNN's requirement for fixed-length inputs, it is uniformly segmented into multiple subsequences, each 2,000 base pairs (bp) long. The total number of subsequences *c* is calculated as:


$$c=\left\lceil \frac{N}{2000}\right\rceil$$ (1)

For sequences whose length is not an integer multiple of 2,000, a zero-padding strategy is applied at the end. Each subsequence is further processed via k-mer segmentation and transformed using the skip-gram model to map each k-mer into a high-dimensional vector space (30). Here, *k* denotes the k-mer length (set to *k* = 3 in this study), and *d* is the number of hidden units in the skip-gram model (set to *d* = 64 in this study). The skip-gram model encodes the contextual relationships between adjacent k-mers as similarities in the vector space, thereby effectively enhancing the ability to capture local patterns. Based on this, each 2,000 bp subsequence can be represented as follows:


$$\begin{array}{l} \text{X}\in {\mathbb{R}}^{\left(2000-k+1\right)\times d}. \end{array}$$ (2)

**X** is the embedding matrix for the subsequences obtained from segmenting the phage and host genome sequences. The row dimension corresponds to the positions of the k-mers within the subsequence, and the column dimension corresponds to the feature dimensions of the skip-gram embeddings.

#### 3.2.2 Local feature learning

The subsequence matrix **X** for the phage and host genome sequences is fed into independent CNN for local feature extraction. The two CNN networks share the same architecture but have independent parameters: CNN_phage_ processes the phage sequences, and CNN_host_ processes the host sequences. Each CNN network consists of three convolutional layers and two pooling operations. Each convolutional layer performs three operations: 2D convolution, batch normalization, and the ReLU activation function [defined as relu(*x*) = max(0, *x*)]. Subsequently, global average pooling is applied to compress the 2D feature matrix into a fixed-length vector. The design of global average pooling offers a dual advantage: it can map input sequences of different lengths into a fixed-dimensional vector, and it has a regularizing effect, helping to mitigate overfitting.

Let $f_{{\text{CNN}}_{\text{phage}}}:X\to {\mathbb{R}}^{256}$ and $f_{{\text{CNN}}_{\text{host}}}:X\to {\mathbb{R}}^{256}$ denote the mapping functions of the respective CNNs, which transform an input subsequence matrix into a 256-dimensional global feature vector (*𝒳* denotes the set of all possible subsequence embedding matrices). The outputs for the *i*-th subsequence are respectively:


$$\begin{array}{ll} {\text{Y}}_{i} & =f_{{\text{CNN}}_{\text{phage}}}\left({\text{X}}_{i}\right)\in {\mathbb{R}}^{256},\;i=1,\ldots ,c \end{array}$$ (3)

$$\begin{array}{ll} {\text{Z}}_{i} & =f_{{\text{CNN}}_{\text{host}}}\left({\text{X}}_{i}\right)\in {\mathbb{R}}^{256},\;i=1,\ldots ,d \end{array}$$ (4)

where *c* and *d* represent the total number of subsequences for the phage and host genomes, respectively, and **Y**_*i*_ and **Z**_*i*_ represent the local feature representations of each subsequence for the phage and host, respectively.

#### 3.2.3 Global dependency learning

To capture long-range dependencies within the sequences, the sequences of local feature vectors extracted by CNN_phage_ and CNN_host_ are fed into two independent Transformer encoders, respectively. Each Transformer encoder consists of a stack of *N* = 6 identical layers. Each layer contains two sub-layers: the first is a multi-head self-attention mechanism, and the second is a simple position-wise fully connected feed-forward network. We employ residual connections around each of the two sub-layers, followed by layer normalization. That is, the output of each sub-layer is LayerNorm[*x*+Sublayer(*x*)], where Sublayer(*x*) is the function implemented by the sub-layer itself. To facilitate these residual connections, all sub-layers in the model, as well as the embedding layers, produce outputs of dimension *d*_model_ = 256.

A learnable CLS vector is prepended to the sequence to aggregate global sequence features. For the phage sequence, the input matrix is $\text{Y}=\left[{\text{Y}}_{\text{CLS}};{\text{Y}}_{1};\ldots ;{\text{Y}}_{c}\right]\in {\mathbb{R}}^{\left(c+1\right)\times 256}$; for the host sequence, the input matrix is $\text{Z}=\left[{\text{Z}}_{\text{CLS}};{\text{Z}}_{1};\ldots ;{\text{Z}}_{d}\right]\in {\mathbb{R}}^{\left(d+1\right)\times 256}$.

The computation of the multi-head self-attention mechanism is as follows. First, the input sequence is linearly projected into query (**Q**), key (**K**), and value (**V**) matrices:


$$\begin{array}{l} \text{Q}=\text{Y}{\text{W}}_{Q},\;\text{K}=\text{Y}{\text{W}}_{K},\;\text{V}=\text{Y}{\text{W}}_{V} \end{array}$$ (5)

where ${\text{W}}_{Q},{\text{W}}_{K},{\text{W}}_{V}\in {\mathbb{R}}^{256\times d_{k}}$ are learnable weight matrices, and *d*_*k*_ is the dimension of each attention head.

The computation for each attention head is:


$$\begin{array}{r} {\text{head}}_{i}=\text{Attention}\left(\text{Q}{\text{W}}_{Q}^{i},\text{K}{\text{W}}_{K}^{i},\text{V}{\text{W}}_{V}^{i}\right)=\\ \mathrm{softmax}\left(\frac{\text{Q}{\text{W}}_{Q}^{i}{\left(\text{K}{\text{W}}_{K}^{i}\right)}^{⊤}}{\sqrt{d_{k}}}\right)\text{V}{\text{W}}_{V}^{i} \end{array}$$ (6)

where ${\text{W}}_{Q}^{i},{\text{W}}_{K}^{i}\in {\mathbb{R}}^{d_{k}\times d_{k}}$, ${\text{W}}_{V}^{i}\in {\mathbb{R}}^{d_{k}\times d_{v}}$ are the projection matrices specific to the *i*-th attention head. This paper uses 4 attention heads (*h* = 4), hence *d*_*k*_ = *d*_*v*_ = *d*_model_/*h* = 256/4 = 64.

The outputs of all attention heads are concatenated and then linearly transformed to produce the final output of the multi-head attention:


$$\begin{array}{l} \text{MultiHead}\left(\text{Y}\right)=\text{Concat}\left({\text{head}}_{1},\ldots ,\text{hea}{\text{d}}_{4}\right){\text{W}}_{O} \end{array}$$ (7)

where ${\text{W}}_{O}\in {\mathbb{R}}^{256\times 256}$ is the output projection matrix. Subsequently, the output is obtained through residual connections and a feed-forward network:


$$\begin{array}{ll} {\text{Y}}^{\prime } & =\text{LayerNorm}\left(\text{Y}+\text{MultiHead}\left(\text{Y}\right)\right) \end{array}$$ (8)

$$\begin{array}{ll} {\text{Y}}^{\prime \prime } & =\text{LayerNorm}\left({\text{Y}}^{\prime }+\text{FFN}\left({\text{Y}}^{\prime }\right)\right) \end{array}$$ (9)

where LayerNorm denotes Layer Normalization and FFN represents the Feed-Forward Network. ${\text{Y}}_{\text{CLS}}^{\prime \prime }\in {\mathbb{R}}^{256}$ and ${\text{Z}}_{\text{CLS}}^{\prime \prime }\in {\mathbb{R}}^{256}$ are the global feature representations for the phage and host sequences, denoted as **C**_phage_ and **C**_host_, respectively.

The phage and host genome sequences undergo the aforementioned CNN and Transformer processing pipeline to obtain their respective global feature vectors **C**_phage_ and **C**_host_.

#### 3.2.4 Fully connected classifier

To predict the specific matching relationship between phages and host bacteria, we construct a fully connected neural network classifier. This classifier takes the global feature vectors of the phage and host as input, learns the interaction patterns between them through multiple layers of nonlinear transformations, and outputs a probability distribution for matching. Specifically, the two global representation vectors are first concatenated to form a comprehensive feature vector:


$$\begin{array}{l} \text{z}=\left[{\text{C}}_{\text{phage}};{\text{C}}_{\text{host}}\right]\in {\mathbb{R}}^{512} \end{array}$$ (10)

This fused feature is then passed through a multilayer perceptron (MLP) for classification prediction, which can be mathematically summarized as:


$$\begin{array}{l} \text{p}=f_{\text{MLP}}\left(\text{z}\right) \end{array}$$ (11)

where *f*_MLP_ denotes a multilayer perceptron with two hidden layers. The first layer expands the input dimension from 512 to 4,096, employing the ReLU activation function to introduce nonlinear transformations. The second layer maps the hidden features to a two-dimensional output space, and the Softmax function converts this into a probability distribution **p**∈ℝ^2^. Here, **p**[0] represents the probability of non-match, and **p**[1] represents the probability of a match. The final prediction is determined by the class with the higher probability.

## 4 Experiments and results analysis

### 4.1 Experiment setup

The model was trained for 200 epochs using the Adam optimizer with a learning rate of 1 × 10^−4^. The exponential decay rates for the moment estimates (β_1_ and β_2_) were set to 0.9 and 0.999, respectively. We employed a mini-batch training strategy with a batch size of 32. To prevent overfitting, an L2 weight decay regularization term with a coefficient of 1 × 10^−3^ was incorporated into the loss function. Furthermore, gradient clipping was applied to mitigate the exploding gradient problem, with the threshold set to 5.0. During testing, the population statistics (mean and variance) used as fixed parameters for normalization were estimated from the training data. These statistics were computed as the moving average of the mini-batch statistics during training, with a decay rate of 0.999.

All experiments were conducted on a workstation equipped with an NVIDIA GeForce RTX 4090 GPU. The deep learning framework was implemented using PyTorch 1.9.0 with CUDA 11.1 support.

### 4.2 Evaluation metrics

To systematically evaluate prediction performance, we followed the standard evaluation protocol in phage-host prediction literature and quantitatively assessed the results across five taxonomic levels: Genus, Family, Order, Class, and Phylum. For any given taxonomic level L, the prediction accuracy is defined as:


$$Accuracy_{L}=\frac{N_{correct,L}}{N_{total}}$$

where *N*_*correct, L*_ represents the number of phages whose predicted host matches the true host at taxonomic level L, and *N*_*total*_ denotes the total number of phage-host pairs in the test set (1,462 in this study). This hierarchical evaluation framework comprehensively measures model performance at different taxonomic resolutions. We placed particular emphasis on Genus-level and Phylum-level accuracy, which reflect the model's capability for fine-grained host prediction and broad taxonomic assignment, respectively. All evaluations were performed on an independent test set that was completely separate from the training data, ensuring unbiased assessment of model generalization capability.

### 4.3 Comparison with existing methods

To comprehensively evaluate the predictive performance of our model, we conducted a systematic comparative analysis against current mainstream phage-host prediction methods on a standardized test set (containing 1,462 phages and 62,493 candidate hosts). The compared methods included representative algorithms such as WIsH (based on Markov models), PHP (which uses k-mer similarity), and VirHostMatcher (VHM) (27). All methods were rigorously evaluated at five major taxonomic levels (Genus, Family, Order, Class, and Phylum) to ensure comprehensive and fair comparison. The experimental results shown in Table 1 fully demonstrate the superior performance of our model. In the most challenging genus-level classification task, our model achieved an accuracy of 36.1%, representing an improvement of 2.1% over PHP's 34.0% and 2.7% over VHM's 33.4%, with this enhancement being statistically significant. As the taxonomic level increases, the prediction accuracy of all methods shows an upward trend, which aligns with the general principle that higher taxonomic levels exhibit stronger sequence conservation. Particularly, our model reached 82.8% accuracy at the phylum level, surpassing PHP's 80.0% by 2.8%, demonstrating the model's stable advantage in high-level classification tasks.

**Table 1:** Performance comparison of different methods across five taxonomic levels (%).

**Method**	**Genus**	**Family**	**Order**	**Class**	**Phylum**
WIsH	33.0	42.0	45.0	69.0	72.0
VHM	33.4	45.0	53.0	70.0	75.0
PHP	34.0	45.0	61.0	78.0	80.0
**Our model**	**36.1**	**48.3**	**65.2**	**81.6**	**82.8**

Bold values indicate the best performance at each taxonomic level.

The performance differences among various methods profoundly reflect the technical characteristics of their underlying algorithmic principles: The WIsH method, based on Markov models, performs well in short sequence prediction tasks but has limited feature extraction capability when processing complete genome sequences; The VirHostMatcher method utilizes k-mer frequency features and enhances prediction performance through multi-feature fusion; The PHP method employs Gaussian models to process k-mer features and demonstrates good adaptability at higher taxonomic levels; In contrast, our model innovatively introduces deep learning technology into the field of phage-host prediction, effectively capturing local sequence patterns through CNN networks, combined with Transformer architecture to model long-range dependencies, achieving end-to-end learning from raw sequences to prediction outcomes, thereby avoiding the limitations of manual feature design in traditional methods and consequently demonstrating significant advantages across multiple taxonomic levels.

### 4.4 Ablation experiment

To validate the effectiveness of each key component in our model, we conducted a series of ablation experiments on the testset (1,462 phages, 62,493 candidate hosts). All experiments used the identical training set and hyperparameter settings. The results across five taxonomic levels (Genus, Family, Order, Class, and Phylum) are shown in Table 2.

**Table 2:** Accuracy comparison of ablation studies across five taxonomic levels (%).

**Model variant**	**Genus**	**Family**	**Order**	**Class**	**Phylum**
**Complete model (ours)**	**36.1**	**48.3**	**65.2**	**81.6**	**82.8**
Skip-gram + CNN (without transformer)	31.5	43.7	60.6	77.0	78.2
CNN + Transformer (without skip-gram)	32.0	44.2	61.1	77.5	78.7

Bold values indicate the best performance at each taxonomic level.

The ablation experiments reveal distinct contributions from each architectural component: The Skip-gram + CNN combination achieved 31.5% accuracy at the genus level, demonstrating that local pattern extraction through convolutional networks combined with semantic embeddings provides a solid foundation for sequence representation. However, the absence of Transformer encoding resulted in a 4.6% drop compared to the complete model, highlighting the importance of global dependency modeling for capturing long-range interactions in genomic sequences. The performance of CNN + Transformer (without Skip-gram) (32.0% at genus level) indicates that while the CNN-Transformer pipeline can learn meaningful representations from raw sequences, the pre-trained semantic embeddings from Skip-gram provide additional discriminative power. The 4.1% improvement when incorporating Skip-gram embeddings demonstrates their value in capturing biological context and k-mer semantics.

## 5 Conclusion

In this paper, we propose a deep Siamese network framework that integrates both local and global features for precise phage selection in pulmonary infections. The framework employs identical model architectures to process phage and host genomes simultaneously. First, k-mer processing captures contextual information from local genomic sequences. Then, CNN and Transformers are applied to extract local and global features, respectively. These features are subsequently integrated to predict phage–host interactions. Experimental results on a dataset created from the NCBI genome database confirm that our method achieves superior performance in accurately identifying phages that target specific bacterial hosts, demonstrating its potential for supporting phage therapy in pulmonary infection treatment.

Although our proposed framework demonstrates superior performance in predicting phage-host interactions, several aspects warrant further improvement. Firstly, while the current model primarily relies on sequence features, future work could integrate multi-omics data such as protein structures and epigenetic profiles. Secondly, the feature interaction mechanism should be further optimized to handle sequence pairs with significantly divergent lengths.

## Data Availability

The original contributions presented in the study are included in the article/supplementary material, further inquiries can be directed to the corresponding authors.
